# Correlations Between Parental Lines and *Indica* Hybrid Rice in Terms of Eating Quality Traits

**DOI:** 10.3389/fnut.2020.583997

**Published:** 2021-01-07

**Authors:** Yan Peng, Bigang Mao, Changquan Zhang, Ye Shao, Tianhao Wu, Liming Hu, Yuanyi Hu, Li Tang, Yaokui Li, Bingran Zhao, Wenbang Tang, Yinghui Xiao

**Affiliations:** ^1^College of Agronomy, Hunan Agricultural University, Changsha, China; ^2^State Key Laboratory of Hybrid Rice, Hunan Hybrid Rice Research Center, Changsha, China; ^3^Longping Graduate School, Hunan University, Changsha, China; ^4^Jiangsu Key Laboratory of Crop Genomics and Molecular Breeding, Jiangsu Key Laboratory of Crop Genetics and Physiology, Jiangsu Co-Innovation Center for Modern Production Technology of Grain Crops, College of Agriculture, Yangzhou University, Yangzhou, China

**Keywords:** eating quality, hybrid rice, parental lines, physicochemical character, starch molecular structure

## Abstract

In this study, by analyzing the relationship between hybrid combinations and parental lines, we found that the eating quality traits of hybrid combinations were determined by both parents. The sterile lines determined the overall eating quality characteristics of the hybrid combinations. For the same sterile line, there were some correlations between the hybrid combinations and restorer lines in terms of taste value, rapid visco analyzer breakdown and setback values, apparent amylose content, and cooked rice hardness and stickiness. Analysis of the starch fine structure between hybrid combinations and their restorer lines demonstrated positive correlations between them in terms of short-branch amylopectin chains and amylose. Moreover, different allelic combinations of the *Wx* gene showed different genetic effects on the eating quality traits of hybrid rice. Overall, this study provides a framework for the development of hybrid rice with superior eating quality.

## Introduction

Rice is a highly important crop that has achieved great success in heterosis applications. Considerable attention has been devoted to the development of high-yield hybrid rice (1–3). As the standard of living continues to improve, consumer preferences are shifting toward rice varieties with superior eating quality. Thus, breeding rice varieties with good eating quality has become a priority. Until now, much progress has been made toward enhancing the eating quality of conventional rice. However, hybrid rice, which is a segregated population of F_1_ hybrids, substantially differs from conventional rice. Therefore, the hybrid rice we consume is a varietal mixture, and it may be inappropriate to evaluate its eating quality using the standards previously established for conventional rice. In addition, as the eating quality of hybrid rice is affected by both parents, by evaluating the quality traits of hybrid combinations from different types of parental lines, we can derive the most appropriate phenotype/genotype combination to achieve high quality.

Starch is the main storage component of the rice endosperm and is the key determinant of rice grain quality and eating quality (4). The apparent amylose content (AAC), gel consistency (GC), and gelatinization temperature (GT) are three key factors affecting the eating and cooking qualities (ECQ) of rice grains (5). The characteristic value of the rapid visco analyzer (RVA) profile has also become an important physicochemical index of rice eating quality (6–8). Starch is a branched glucose polymer comprising amylopectin and amylose. These molecules constitute the semi-crystalline structure of starch grains (4, 9). Earlier studies demonstrated that the molecular size of amylose, the proportion of its branches, and the structure of amylopectin significantly influence cooked rice texture (10, 11). Variations in the amylopectin structure contribute to the differences in quality among rice varieties with similar amylose content (12, 13). The proportion of short amylopectin chains greatly influences rice taste (14). Therefore, the proportions of amylose and amylopectin and the fine structure of the latter should be considered in the endeavor to improve hybrid rice quality.

So far, numerous studies have examined the physicochemical properties and molecular structure of starch in conventional rice (15–21). However, relatively little research has investigated hybrid rice (22–24). Moreover, we found no reports on the correlations between hybrid rice and parental lines in terms of the aforementioned characteristic indices. Therefore, this study aimed to (i) determine the physicochemical properties and starch molecular structures related to the eating quality of hybrid rice, (ii) examine the relationships between hybrid combinations and parental lines in terms of these parameters, and (iii) analyze the genetic effects of different allelic combinations of *Wx* or *ALK* on the eating quality traits of hybrid rice. Our results will provide a theoretical basis for improving the eating quality of *indica* hybrid rice.

## Materials and Methods

### Materials

The rice accessions used in this study included 400 hybrid combinations (F_2_) and 80 parental lines, all of which were frontier breeding materials from the College of Agronomy, Hunan Agricultural University (China) and the Yuan Longping High-tech Agriculture Co., Ltd. (China). In addition, 12 representative materials, which were collected from the Hunan Hybrid Rice Research Center (China), were used for correlation analysis between sensory evaluation and taste value determined by the rice taste analyzer. Among these 12 materials, Meixiangzhan 2, Xiangwanxian 13, Xiangwanxian 17, Xiangyaxiangzhan, and Yuewangsimiao (A1–A6), are representative indica conventional rice with high eating quality in South China. Luliangyou 996, Zhuliangyou 819, Cliangyou 343, Yliangyou 1, Jingliangyou 534, and Tianyouhuazhan (A7–A12) are six representative indica hybrid rice varieties with high yields but different eating qualities in South China. All varieties were planted in the same field on an experimental farm in Chunhua town, Changsha, China (28°2592" N, 113°2311" E). The planting season was from June 3 to October 3. Each hybrid rice line was planted in six rows per plot and six plants per row. The seeds were harvested from 20 mature plants in the middle of each plot, thoroughly mixed, and allowed to air-dry at room temperature (15–25°C) for 2 months.

### Flour and Starch Preparation

The mature seeds were de-husked with a rice huller (SY88-TH, Korea) and milled with a grain polisher (Kett, Tokyo, Japan). A portion of the polished rice samples was stored in sealed bags at 4°C for later use in taste evaluations. The remaining polished rice was ground into flour in a mill (FOSS 1093 Cyclotec Sample Mill; Foss A/S, Hillerød, Denmark) and passed through a 100-mesh sieve.

### Taste Value

A rice taste meter (model STA1B, Sasaki Company) was used to determine the taste values of all hybrid rice and parental lines. First, 30 g of milled rice was placed in a stainless steel sink containing water and soaked for 30 min. The rice was rinsed three times in cold water, and sufficient water was replenished to achieve a 1:1.4 mass ratio of rice to water. The rice was cooked for 45 min, kept warm for 10 min, and cooled to room temperature for ≤2 h. The rice taste meter included three lines of measurement: Japanese rice, China *japonica* rice, and *China indica* rice. As all varieties were *indica* hybrid rice or *indica* rice, we chose the China *indica* rice measuring line for the taste evaluation. The taste value mainly reflects the appearance (glossiness and whiteness) of cooked rice: <50, very poor; 50–60, poor; 60–70, mediocre; 70–80, good; 80–100, very good. The high-quality *indica* rice line Yuzhenxiang and *indica* hybrid rice line Jingliangyou 534 were used as controls. Each sample was measured three times, and the average was calculated and recorded.

### Sensory Evaluation

The sensory evaluation was conducted according to GB/T 15682-2008 published by the Ministry of Agriculture of China. The sensory evaluation team comprised eight people of different genders and ages who could professionally identify taste. Four samples were evaluated at a time, including one reference sample (Huang Huazhan, with moderate eating quality) and three samples to be evaluated. The tasting evaluation included fragrance, appearance, palatability (viscosity, hardness, and elasticity), taste, and cold rice texture. The comprehensive scores were as follows: ≤50, very poor; 51–60, poor; 61–70, average; 71–80, relatively good; 81–90, good; and >90, very good.

### Physicochemical Analyses

Grain shape and chalkiness were measured with a ScanMaker grain appearance analyzer (WSeen SC-E; Hangzhou WSeen Detection Technology Co. Ltd., Hangzhou, China). The apparent amylose content and gel consistency were measured according to the method of (25). The protein content was measured by near-infrared spectroscopy. The viscosity was determined using an RVA (Newport Scientific PTY Ltd., Warriewood, Australia) (26). The cooked rice texture was evaluated with a texture analyzer (SATAKE RHS1A; Satake Corp., Hiroshima, Japan). The thermal properties were measured by differential scanning calorimetry (DSC200F3; Netzsch Instruments NA LLC, Burlington, MA, USA) (27). All tests were performed in triplicate.

### Alkali Spreading Value (ASV)

Eighteen intact milled hybrid rice grains were immersed in 1.7% (w/v) KOH and incubated at 30°C for 24 h. Grain dispersity was then observed, and the ASV for each hybrid combination was calculated according to Mariotti et al. (28).

### Gel Permeation Chromatography (GPC)

Purified rice starch was debranched with isoamylase (EC3.2.1.68, E-ISAMY; Megazyme, Bray, Ireland) and dissolved in dimethyl sulfoxide. The relative molecular weight distribution of the debranched starch was determined by GPC in a PLGPC 220 system (Polymer Laboratories Varian, Inc., Amherst, MA, USA). GPC data were transformed, and molecular weight distribution curves were plotted as described by Zhang (29). All tests were performed in triplicate.

### Analysis of the Wx and ALK Allelic Variation

Genomic DNA was extracted from the fresh leaves of all parental lines using a modified CTAB method. The *Wx* and *ALK* allelic variations were analyzed by KASP genotyping (24) and Sanger sequencing. KASP genotyping: the allele-specific primers were designed to carry the standard FAM (5-GAAGGTGACCAAGTTCATGCT-3) and HEX (5 GAAGGTCGGAGTCAACGGATT 3) tails and the targeted SNP at the 3 end. KASP 1-*Wx*^*a*^/*Wx*^*b*^ (F1: 5′-*GAAGGTGACCAAGTTCATGCT*TTCATCAGGAAGAACATCTGCAAGG-3; F2: 5 *GAAGGTCGGAGTCAACGGATT*TTCATCAGGAAGAACATCTGCAAGT-3; R1: 5′- GCCCAACACCTTACAGAAATTAGCA-3′); KASP 2-*Wx*^*lv*^/*Wx*^*a*^ (F3: 5′-*GAAGGTGACCAAGTTCATGCT*CTGGAGGAACAGAAGGGCC-3′; F4: 5 *GAAGGTCGGAGTCAACGGATT*CTGGAGGAACAGAAGGGCT-3′; R2: 5-GAAGAACGATCTGGACGTCCTC-3′). Assays were carried out in 384-well formats and 10-μL reactions (20–30 ng/μL DNA, 5 μL 1 × KASP master mixture, 0.14 μL KASP assay mix, and 4.86 μL water). PCR was conducted using the following protocol: hot start at 94°C for 15 min, ten touchdown cycles (94°C for 20 s, initial touchdown at 61°C, and then a decrease of −0.6°C per cycle for 60 s), and 26 additional cycles of annealing (94°C for 20 s, 55°C for 60 s). Finally, the PCR product with fluorescent labeling was scanned using a Roche Light Cycler 480 (37°C for 1 min) (24). Sanger sequencing: A 529-bp sequence containing the polymorphic sites was amplified using the primer pair 5- GGGCAGAAAGGTGTGGACATCAT-3 and 5- ACCATTGGTACTTGGCCTTGACA-3. PCR amplification was as follows: initial DNA denaturation at 95°C for 4 min; 30 cycles of denaturation at 95°C for 30 s, annealing at 58°C for 30 s, and extension at 72°C for 30 s; and final extension at 72°C for 5 min. After gel purification, the PCR products were sequenced by TsingKe Biology Technology (Beijing, China).

### Statistical Analysis

The experiments were carried out in triplicate, and the data were reported as mean values and standard deviations (±SD). One-way ANOVA and a Tukey's multiple-comparison test were used to determine significant differences among the mean values by using the SPSS 16.0 statistical software program.

## Results

### Taste Values of Hybrid Rice and Their Parental Lines

**Figure 1:**
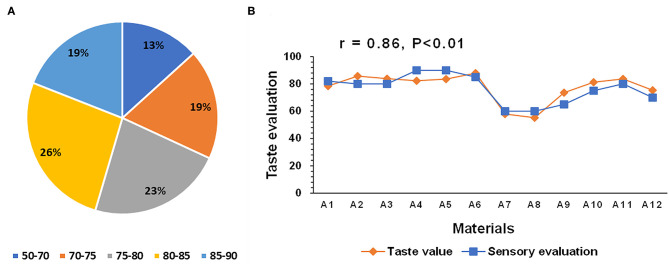
Taste values of 400 hybrid rice varieties and 80 parental lines **(A)** and correlation between the sensory evaluation and taste value determined by a rice taste analyzer **(B)**. **(A)** Different colors represents different taste value ranges. Percentage in each fan-shaped region represents the proportion of each taste value range. **(B)** Materials A1–A6 are conventional indica rice varieties. Materials A7–A12 are hybrid rice varieties.

The taste values of 400 hybrid rice and 80 parental lines were measured using the *indica* rice standard of the rice taste analyzer. The comprehensive taste value scores of the hybrid combinations ranged between 50–90. All hybrid combinations were categorized into five taste value levels with proportions of 13%, 17%, 22%, 27%, and 21% (Figure 1A). A series of conventional *indica* and hybrid rice varieties were used for correlation analysis between the sensory evaluation and taste value. The overall trend of the sensory evaluation was consistent with that of the taste value, with a correlation of 0.86 (Figure 1B). Thus, the rice taste analyzer can feasibly assess hybrid rice eating quality.

### Physicochemical Properties of Hybrid Rice and Their Parental Lines

We determined the AAC, GC, GT, RVA viscosity parameters, ASV, protein content, cooked rice hardness and stickiness, and grain shape and chalkiness of the hybrid rice and parental lines. Supplementary Table 1 shows the statistical values of the physicochemical properties of all lines.

### Correlation Between the Physicochemical Properties and Taste Value of Hybrid Rice

We analyzed the correlation between the physicochemical properties and taste values of hybrid rice. Supplementary Table 2 shows that the taste value of the hybrid rice was negatively correlated with the AAC, protein content, setback value (SBV), final viscosity of the RVA profile (CPV), and hardness of the cooked rice grain. Moreover, it was positively correlated with the gel consistency, breakdown value (BDV), peak viscosity of the RVA profile (PKV), and stickiness of the cooked rice grain. These findings were consistent with those reported for conventional rice in previous studies (7, 30, 31). However, there were no obvious correlations between taste value and grain shape or chalkiness.

### Effect of GT on the Eating Quality of Hybrid Rice

**Figure 2:**
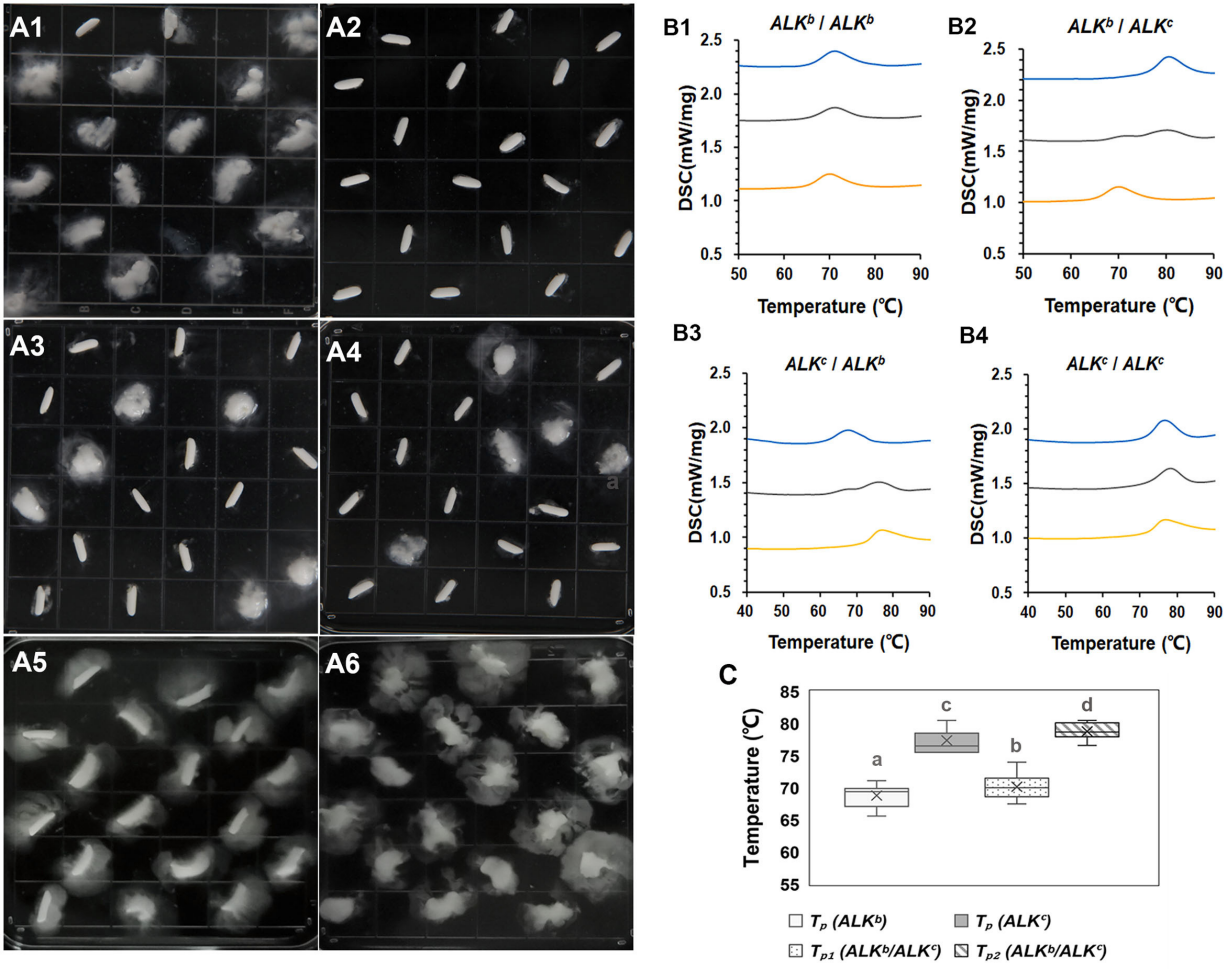
Alkali spreading value and DSC thermograms of hybrid combinations with various allelic combinations of *ALK*. **(A1–A4)** show the alkali spreading values of the hybrid combinations with *ALK*^*b*^/*ALK*^*b*^, *ALK*^*c*^/*ALK*^*c*^, *ALK*^*c*^/*ALK*^*b*^, and *ALK*^*b*^/*ALK*^*c*^, respectively. **(A5,A6)** show the alkali spreading values of a *japonica* control (*ALK*^*b*^/*ALK*^*b*^) and an *indica* control (*ALK*^*c*^/*ALK*^*c*^), respectively. **(B1–B4)**: orange, blue, and gray lines represent sterile lines, restored lines, and hybrid combinations, respectively. **(C)**: Show the *T*_p_ value of hybrid rice with different *ALK* allelic combinations. *T*_p1_ and *T*_p2_ are the peak temperatures of peak 1 and peak 2 in the hybrids with different allelic combinations, different superscripted letters on the boxplots indicate significant differences (*P* < 0.05).

We found that the hybrid rice grains had distinct and separate ASVs (Figure 2A). Thus, DSC was used to analyze GT variation in the hybrid rice. Figure 2B shows two-peak thermal curves for most of the hybrid rice varieties, especially when there were obvious differences in GT between the parental lines of the hybrid combination. This finding was consistent with the ASV results. *ALK* (*SSIIa*) is the major gene controlling GT, and allelic variation in *ALK* is responsible for natural variations in GT (32, 33). Varieties carrying *ALK*^*c*^ have higher GT values than those carrying *ALK*^*b*^ (34). Analysis of the allelic variations in *ALK* among the varieties indicated that *ALK*^*c*^ and *ALK*^*b*^ are common in hybrid rice. Therefore, we examined the ASVs and DSC thermograms of hybrid rice with the allelic combinations *ALK*^*b*^/*ALK*^*b*^, *ALK*^*c*^/*ALK*^*c*^, *ALK*^*b*^/*ALK*^*c*^, and *ALK*^*c*^/*ALK*^*b*^. The ASV analysis revealed no clear differences in gelatinization among hybrid rice grains with the same *ALK* allelic combinations (*ALK*^*b*^/*ALK*^*b*^ and *ALK*^*c*^/*ALK*^*c*^); however, obvious differences in gelatinization among hybrid rice grains with different allelic combinations (*ALK*^*b*^/*ALK*^*c*^ and *ALK*^*c*^/*ALK*^*b*^) were seen (Figures 2A1–A4). The DSC thermograms showed that hybrid rice with the same *ALK* allelic combinations (*ALK*^*b*^/*ALK*^*b*^ and *ALK*^*c*^/*ALK*^*c*^) exhibited a single peak, whereas those with different allelic combinations (*ALK*^*b*^/*ALK*^*c*^ and *ALK*^*c*^/*ALK*^*b*^) presented distinct two-peak thermal curves (Figures 2B1–B4). The relationship between the hybrid combinations and parental lines in terms of peak temperature (*T*_p_) was also evaluated. Supplementary Table 3 shows that most hybrid combinations with single thermal curves had a relatively lower *T*_p_ when both parents carried *ALK*^*b*^. In contrast, they had a comparatively higher *T*_p_ if both parents carried *ALK*^*c*^. For most hybrids (*ALK*^*b*^/*ALK*^*c*^ and *ALK*^*c*^/*ALK*^*b*^) with two-peak thermal curves, those with parents carrying the *ALK*^*b*^ allele showed peak 1 ≥ *T*_p_. However, peak 2 ≥ *T*_p_ was observed for those with parents carrying the *ALK*^*c*^ allele. As the GTs and ASVs of the hybrid rice combinations could not be accurately measured, correlation analysis between taste value and pasting temperature of RVA were analyzed. However, we detected no obvious correlation between these parameters.

### Physicochemical Relationship Between Hybrid Combinations and Parental Lines

**Figure 3:**
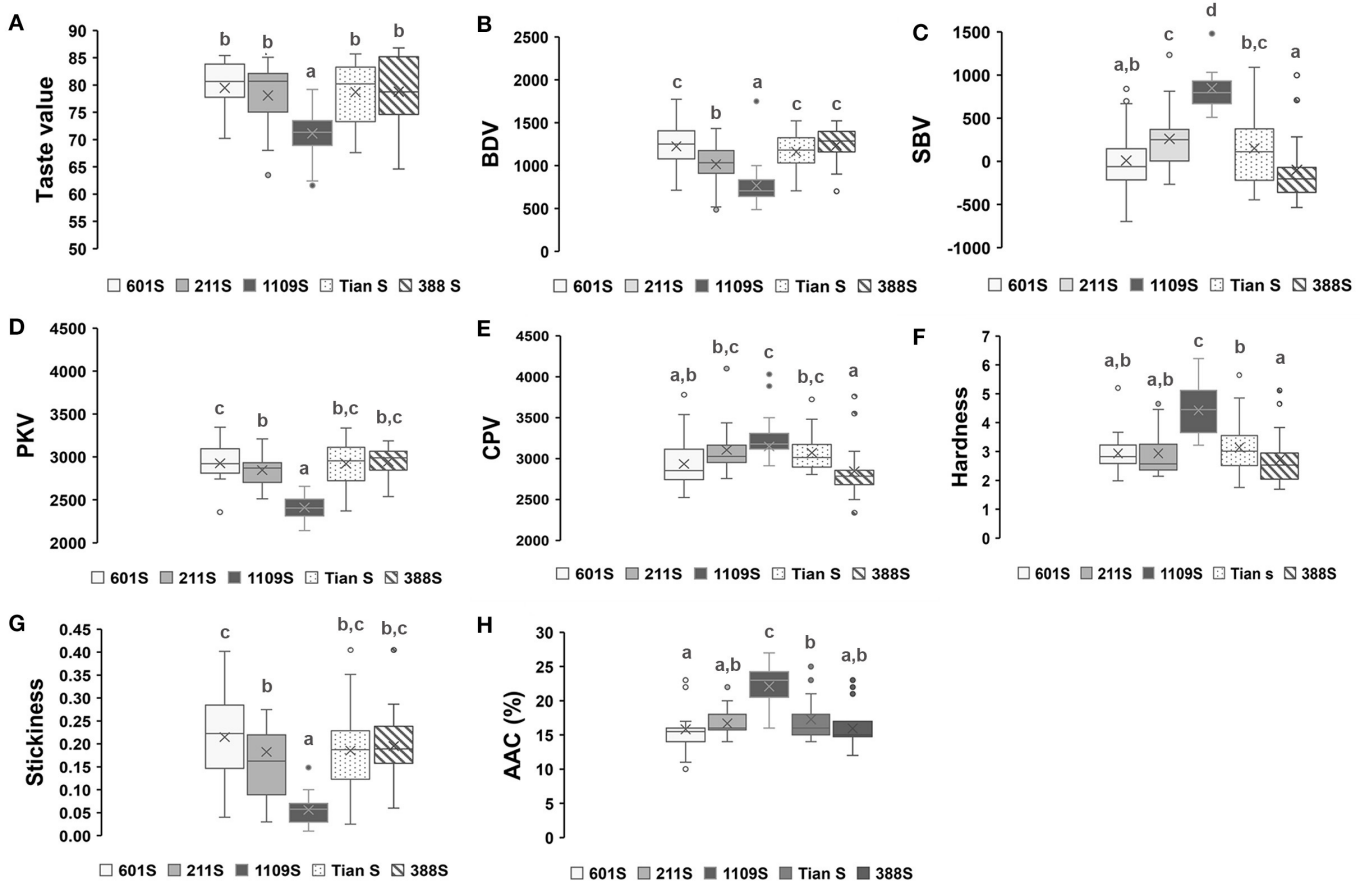
Boxplots of taste value and physicochemical properties of hybrid combinations. **(A–H)** Boxplots of taste value and physicochemical properties of hybrid combinations derived from five sterile lines and 22 restored lines, different superscripted letters on the boxplots indicate significant differences (*P* < 0.05).

In this study, certain hybrid combinations with different taste values were obtained by crossing the same sterile line with various restorer lines. Hence, five sets of materials were selected to analyze the physicochemical relationship between the hybrid combinations and their parental lines (Figure 3). Each group included a sterile line, 22 restorer lines, and 22 hybrid combinations; the restorer lines were the same for all five groups. The taste values and physicochemical properties of all hybrids and parental lines are shown in Appendix 1.

As the sterile line for the first group, 1109S presented the lowest taste value and viscosity but the highest AAC (26%). Most hybrid combinations derived from it had comparatively lower taste values, breakdown values, and cooked rice stickiness but higher setback values, AAC, and cooked rice hardness. Sterile lines 601S and Tian S had relatively higher taste values and RVA breakdown but showed lower RVA setback values and AAC (13%–16%), except for some combinations, which were bred by crossing the same sterile line and certain restorer lines with higher setback values and AAC but very low breakdown values and taste values. All other hybrid combinations derived from the aforementioned sterile lines presented relatively high breakdown values and cooked rice stickiness but comparatively low setback values, AAC, and cooked rice hardness. The sterile line of the fifth group, 211S, exhibited intermediate taste value, AAC (18%), RVA profile breakdown, and setback values. The hybrid combinations derived from 211S had roughly equal numbers of every taste grade. The sterile 388S line had relatively lower taste values and breakdown values but comparatively higher setback values and AAC (23%). In these respects, it resembled the 1109S line; however, the taste values were relatively higher for most hybrid combinations in this group. A comparison of the physicochemical properties of the two sterile lines revealed that 1109S showed a slightly higher AAC but a lower RVA curve, which was similar to that of the *indica* cultivar Q11 carrying the *Wx*^*lv*^ allele (35). In general, the above results indicated that the sterile lines determined the overall eating quality characteristic levels of hybrid rice. Therefore, in future quality breeding programs of hybrid rice, sterile lines similar to 1109S should be removed, while those similar to Tian S and 601S should be used effectively.

Since each group included a sterile line, 22 restorer lines, and 22 hybrid combinations, we performed Pearson correlation analysis on the physicochemical properties of the hybrid combinations and corresponding restorer lines in each group (Table 1). For the same sterile line among these groups, the hybrid combinations and their restorer lines were positively correlated in terms of taste value. Except for the 1109S line, there were some correlations between the hybrid combinations and restorer lines in terms of AAC, RVA breakdown, and setback values. Supplementary Figure 1 shows the RVA profile between the parental lines and hybrid combinations with different taste values. Overall, the hybrid combinations with higher taste values had higher RVA breakdown and lower RVA setback values than the hybrid combinations with lower taste values. This was consistent with the corresponding restorer lines in the same group.

**Table 1:** Correlation coefficients of physicochemical properties and taste values between hybrid combinations and restorer lines.

**Sterile line**	**Restorer** **Hybrids**	**Taste value**	**BDV**	**SBV**	**PKV**	**CPV**	**Hardness**	**Stickiness**	**AAC**
601 S	Taste value	0.52^*^	0.48^*^	−0.51^*^	0.13	−0.58^**^	−0.51^*^	0.49^*^	−0.55^**^
	BDV	0.26	0.46^*^	−0.49^*^	0.10	−0.59^**^	−0.22	0.35	−0.37
	SBV	−0.20	−0.42^*^	0.42	−0.14	0.46^*^	0.22	−0.25	0.32
	PKV	0.23	0.27	−0.32	−0.07	−0.49^*^	0.06	0.15	−0.34
	CPV	−0.19	−0.27	0.28	−0.21	0.21	0.39	−0.27	0.11
	Hardness	−0.54^**^	−0.46^*^	0.54^**^	−0.13	0.64^**^	0.50^*^	−0.50^*^	0.63^**^
	Stickiness	0.54^**^	0.60^**^	−0.65^**^	0.34	−0.61^**^	−0.45^*^	0.65^**^	−0.45^*^
	AAC	−0.45^*^	−0.55^**^	0.60^**^	−0.26	0.61^**^	0.48^*^	−0.51^*^	0.56^**^
211 S	Taste value	0.53^*^	0.35	−0.35	0.24	−0.29	−0.70^**^	0.20	−0.72^**^
	BDV	0.69^**^	0.68^**^	−0.68^**^	0.54^**^	−0.52^*^	−0.67^**^	0.24	−0.70^**^
	SBV	−0.52^*^	−0.44^*^	0.51^*^	−0.38	0.40	0.50^*^	−0.16	0.56^**^
	PKV	0.48^*^	0.46^*^	−0.46^*^	0.33	−0.38	−0.29	0.08	−0.41
	CPV	−0.22	−0.19	0.20	−0.31	0.05	0.42	−0.11	0.41
	Hardness	−0.39	−0.27	0.27	−0.26	0.17	0.61^**^	−0.17	0.67^**^
	Stickiness	0.50^*^	0.37	−0.44^*^	0.55^**^	−0.22	−0.60^**^	0.24	−0.71^**^
	AAC	−0.51^*^	−0.49^*^	0.50^*^	−0.41	0.38	0.78^**^	−0.45^*^	0.79^**^
1109 S	Taste value	0.65^**^	0.42^*^	−0.07	0.16	−0.11	−0.18	0.31	−0.06
	BDV	0.57^**^	0.16	−0.10	0.25	0.16	−0.03	0.29	−0.40
	SBV	−0.61^**^	−0.09	0.22	−0.10	−0.11	0.10	−0.28	0.21
	PKV	0.20	0.04	−0.24	0.29	0.12	−0.02	0.09	−0.46^*^
	CPV	−0.49^*^	−0.28	−0.07	0.04	0.07	0.09	−0.14	−0.20
	Hardness	−0.70^**^	−0.44^*^	0.23	−0.12	0.22	0.21	−0.45^*^	0.15
	Stickiness	0.57^**^	0.42	−0.05	0.12	−0.09	−0.08	0.43^*^	−0.13
	AAC	−0.70^**^	−0.30	0.09	−0.11	0.04	0.29	−0.43^*^	0.13
Tian S	Taste value	0.72^**^	0.70^**^	−0.82^**^	0.59^**^	−0.83^**^	−0.64^**^	0.67^**^	−0.87^**^
	BDV	0.65^**^	0.57^**^	−0.69^**^	0.66^**^	−0.54^*^	−0.51^*^	0.57^**^	−0.64^**^
	SBV	−0.57^**^	−0.50^*^	0.59^**^	−0.63^**^	0.38	0.45^*^	−0.57^**^	0.55^**^
	PKV	0.17	0.30	−0.41	0.30	−0.42	−0.10	0.34	−0.31
	CPV	−0.64^**^	−0.38	0.44^*^	−0.53^*^	0.22	0.55^**^	−0.39	0.45^*^
	Hardness	−0.64^**^	−0.66^**^	0.75^**^	−0.47^*^	0.82^**^	0.72^**^	−0.54^**^	0.89^**^
	Stickiness	0.78^**^	0.74^**^	−0.80^**^	0.75^**^	−0.62^**^	−0.55^**^	0.73^**^	−0.72^**^
	AAC	−0.78^**^	−0.72^**^	0.83^**^	−0.72^**^	0.71^**^	0.73^**^	−0.69^**^	0.85^**^
388S	Taste value	0.59^**^	0.75^**^	−0.82^**^	0.71^**^	−0.66^**^	−0.59^**^	0.43^*^	−0.64^**^
	BDV	0.56^**^	0.62^**^	−0.69^**^	0.39	−0.65^**^	−0.62^**^	0.38	−0.66^**^
	SBV	−0.26	−0.38	0.46^*^	−0.27	0.43^*^	0.22	−0.12	0.53^*^
	PKV	0.22	0.30	−0.38	0.02	−0.44^*^	−0.30	0.11	−0.43^*^
	CPV	−0.26	−0.35	0.38	−0.39	0.28	0.19	−0.18	0.36
	Hardness	−0.52^*^	−0.53^*^	0.64^**^	−0.58^**^	0.50^*^	0.40	−0.31	0.54^**^
	Stickiness	0.58^**^	0.51^*^	−0.59^**^	0.50^*^	−0.48^*^	−0.56^**^	0.44^*^	−0.54^**^
	AAC	−0.50^*^	−0.56^**^	0.70^**^	−0.57^**^	0.58^**^	0.47^*^	−0.45^*^	0.70^**^

* Correlations significant at P < 0.05;

** *Correlations significant at P < 0.01*.

### Genetic Effects of Different Allelic Combinations of Wx or ALK on the Eating Quality Traits of Hybrid Rice

**Figure 4:**
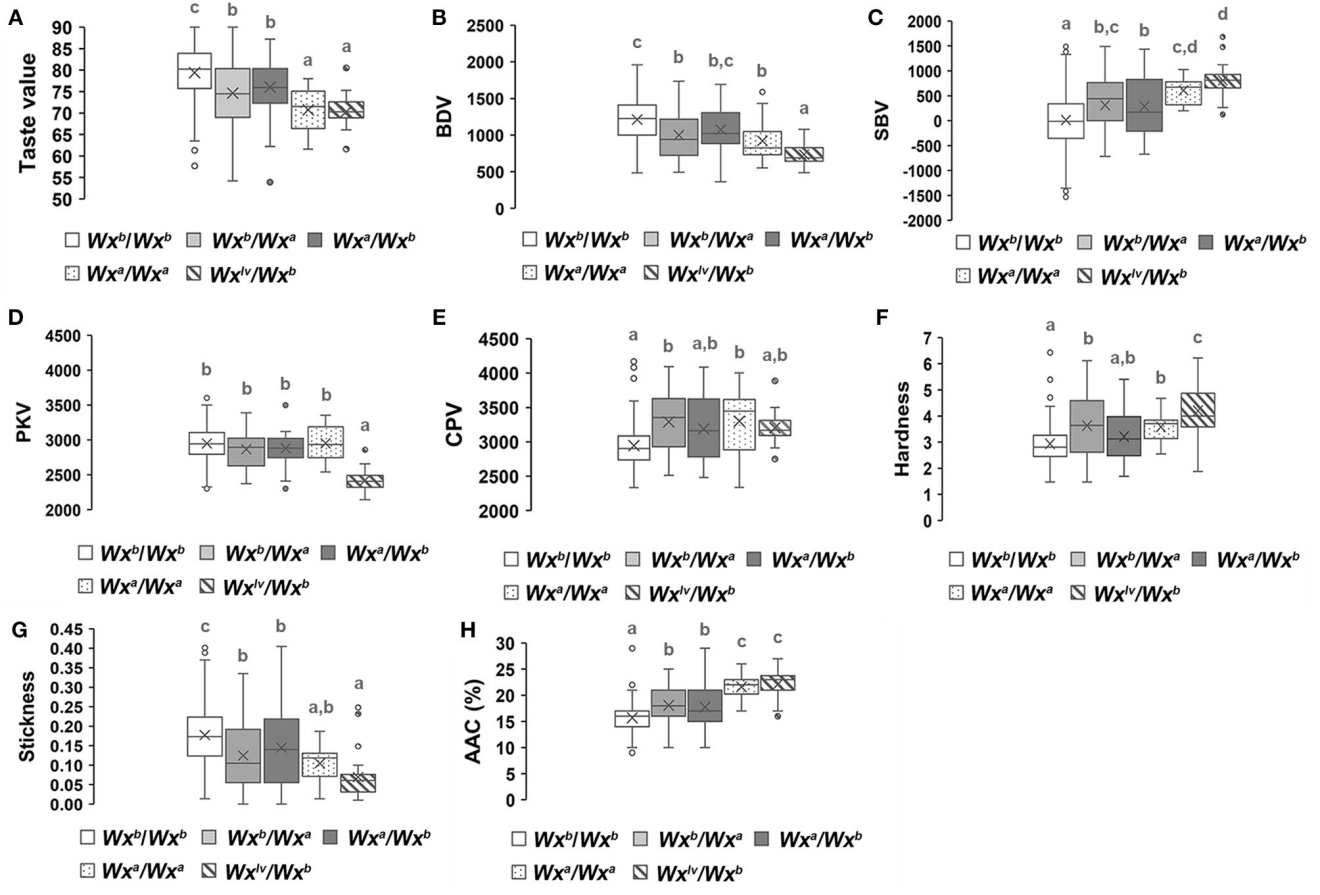
Genetic effects of different allelic combinations of the *Wx* gene on the eating quality traits of hybrid rice. A white box represents *Wx*^*b*^/*Wx*^*b*^, a light gray box represents *Wx*^*b*^/*Wx*^*a*^, a charcoal gray box represents *Wx*^*a*^/*Wx*^*b*^, a box with spots represents *Wx*^*a*^/*Wx*^*a*^, and a box with a slash (\) represents *Wx*^*lv*^/*Wx*^*b*^, different superscripted letters on the boxplots indicate significant differences (*P* < 0.05).

**Figure 5:**
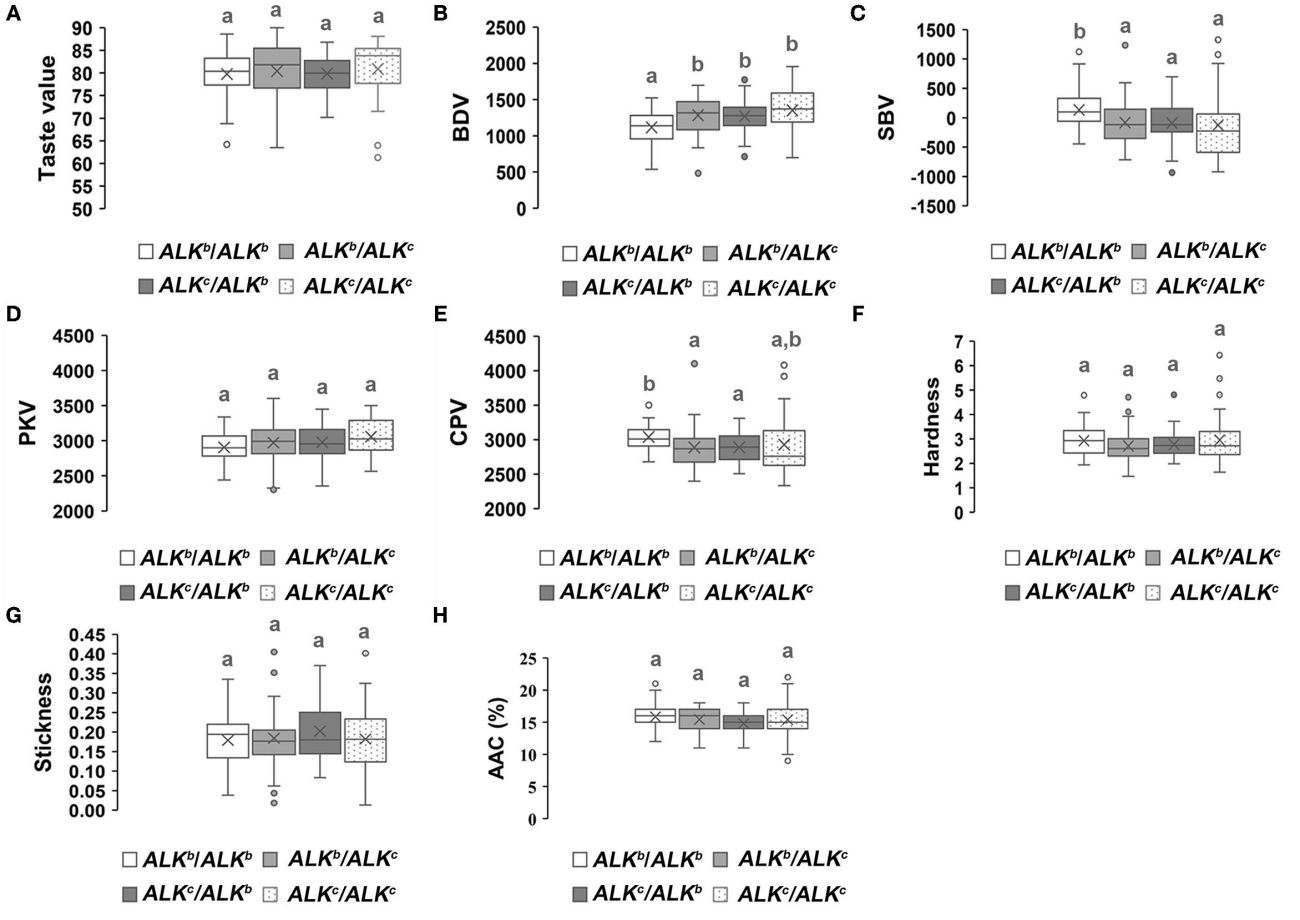
Genetic effects of different allelic combinations of the *ALK* gene on the eating quality traits of hybrid rice. A white box represents *ALK*^*b*^/*ALK*^*b*^, a light gray box represents *ALK*^*b*^/*ALK*^*c*^, a charcoal gray box represents *ALK*^*c*^/*ALK*^*b*^, and a box with spots represents *ALK*^*c*^/*ALK*^*c*^, different superscripted letters on the boxplots indicate significant differences (*P* < 0.05).

We analyzed the allelic variation of *Wx* and *ALK* among all parental lines (Supplementary Figure 2). Three *Wx* (*Wx*^*a*^*, Wx*^*b*^, and *Wx*^*lv*^) and two *ALK* (*ALK*^*b*^ and *ALK*^*c*^) allelic variations were found in the parental lines. Furthermore, the genetic effects of different allelic combinations of *Wx* or *ALK* on the eating quality traits of hybrid rice were analyzed. Figure 4 reveals that the hybrid combinations with *Wx*^*b*^/*Wx*^*b*^ had the highest overall taste values, stickiness, breakdown values, and peak viscosity, while the hardness, AAC, setback values, and final viscosity were lower than those of the other allelic combinations. However, the *Wx*^*lv*^/*Wx*^*b*^ hybrid combinations showed the lowest overall taste values, stickiness, breakdown values, and peak viscosity as well as the highest hardness, AAC, and setback values. The genetic effects of *Wx*^*a*^ / *Wx*^*a*^ were similar to those of *Wx*^*lv*^/*Wx*^*b*^. The overall level of eating quality traits of the *Wx*^*b*^/*Wx*^*a*^ or *Wx*^*a*^/*Wx*^*b*^ hybrid combinations were intermediate. As *Wx*^*b*^ was the main allelic variation of the parental lines, we further analyzed the genetic effects of different allelic combinations of *ALK* in the genetic background of *Wx*^*b*^/*Wx*^*b*^. Hybrid combinations with different allelic combinations of *ALK* showed no obvious difference in the overall taste value, hardness, stickiness, and AAC (Figure 5). However, hybrid combinations with *ALK*^*c*^/*ALK*^*c*^ showed a comparatively higher breakdown value and final viscosity with a comparatively lower setback value, while hybrid combinations with *ALK*^*b*^/*ALK*^*b*^ showed the opposite effects.

### Relative Molecular Weight Distributions of Hybrid Rice and Parental Lines

In total, 21 hybrid combinations with different taste values and 18 parental lines were used to analyze the relative molecular weight distributions (Supplementary Table 4). All hybrid combinations and parental lines showed typical trimodal molecular weight distributions with low, mid, and high peaks corresponding to the short-branch amylopectin chains (AP1), long-branch amylopectin chains (AP2), and amylose (AM) fractions, respectively (36). Supplementary Figure 3 shows that the hybrid combinations and parental lines with higher taste values had relatively fewer AM fractions and greater numbers of amylopectin chains in the AP1 fraction than the hybrids with lower taste values. The AM and AP1 fractions mainly accounted for the substantial differences in taste values among the hybrid rice lines. Correlation analyses between the molecular weight distributions and physicochemical properties in both hybrid combinations and parental lines revealed that AP1 fractions were positively correlated with the taste value, BDV of the RVA profile, and stickiness of the cooked rice grain. However, they were negatively correlated with the SBV of the RVA profile, hardness of the cooked rice grain, and AAC. The AM fractions were negatively correlated with the taste value, BDV of the RVA profile, and stickiness of the cooked rice grain but were positively correlated with the SBV of the RVA profile, hardness of the cooked rice grain, and AAC (Table 2). Correlation analyses also revealed that AP2 fractions were correlated with BDV, SBV of the RVA profile, and texture properties. Moreover, AP2 showed a positive correlation with the taste value in hybrid combinations (*r* = 0.54, *p* < 0.01), but no obvious correlation was observed between these in parental lines. Furthermore, AP1/AP2 were positively correlated with the taste value in parental lines but not in the hybrid combinations.

**Table 2:** Analysis of the correlation between molecular weight distribution and physicochemical properties in both hybrid combinations and parental lines.

	**Parental lines**				**Hybrid combinations**			
	**AP1/AP**	**AP2/AP**	**AM/** **(AM+AP1+AP2)**	**AP1/** **AP2**	**AP1/AP**	**AP2/AP**	**AM/** **(AM+AP1+AP2)**	**AP1/** **AP2**
Taste value	0.96^**^	0.44	−0.96^**^	0.74^**^	0.77^**^	0.54^**^	−0.80^**^	0.40
BDV	0.83^**^	0.64^*^	−0.91^**^	0.49	0.71^**^	0.49^*^	−0.78^**^	0.37
SBV	−0.70^**^	−0.73^**^	0.77^**^	−0.23	−0.78^**^	−0.52^*^	0.84^**^	−0.43^*^
PKV	0.46	0.41	−0.50	0.21	0.50^*^	0.32	−0.53^*^	0.27
CPV	−0.43	−0.39	0.45	−0.18	−0.72^**^	−0.46^*^	0.79^**^	−0.40
Hardness	−0.90^**^	−0.59^*^	0.93^**^	−0.56^*^	−0.83^**^	−0.47^*^	0.87^**^	−0.50^*^
Stickiness	0.90^**^	0.63^*^	−0.91^**^	0.52	0.68^**^	0.67^**^	−0.76^**^	0.25
AAC	−0.91^**^	−0.66^*^	0.94^**^	−0.52	−0.76^**^	−0.48^*^	0.83^**^	−0.42

* Correlations significant at P < 0.05;

** *Correlations significant at P < 0.01*.

In this study, we revealed positive correlations between the hybrid combinations and their restorer lines in terms of their AM and AP1 molecular weight distributions (*r* = 0.61, *p* < 0.01; *r* = 0.66, *p* < 0.01).

## Discussion

Here, we analyzed the physicochemical properties and molecular structures of starch in several hybrid combinations with different taste values. We found that the taste value was correlated with the AAC, RVA peak viscosity, breakdown and setback values, hardness and stickiness of cooked rice, protein content, and GC, which was consistent with the results of previous studies (30, 31). Additionally, taste value was positively correlated with the AP1 fraction and negatively correlated with the AM fraction. These results are consistent with previous reports in conventional rice (15, 36, 37). The influence of the fine structural features of starch on the physicochemical properties of hybrid rice and parental lines was also investigated. Among these structural parameters, AP1 was positively correlated with BDV but negatively correlated with SBV. RVA breakdown is caused by disruption of the gelatinized starch granule structure. Previous reports on conventional rice demonstrated that a higher proportion of short chains in starch would result in high paste breakdown due to greater fragility of the swollen granules (38). The RVA setback value reflects the retrogradation characteristic after cooling. As the temperature decreased, the dissociated amylopectin and amylose were rearranged to form a new amylose-amylopectin network, and a higher proportion of short chains could reduce the reassociation of starch molecules, which may improve the anti-aging performance of rice starch (39). Although previous studies indicated that nearly all of the amylose leached out when a starch-water suspension was heated to the peak viscosity of RVA (40), the present results also showed that the AM fraction was positively correlated with SBV but negatively correlated with BDV. We speculate that this could be caused by other factors affecting RVA characteristics, such as lipids, protein structure, and composition. Our data also showed that the AP1 fraction was positively correlated with stickiness but negatively correlated with hardness, while the AM fraction was negatively correlated with stickiness but positively correlated with hardness. Amylose-lipid complexes can form an insoluble layer around the granules, preventing the entry of water. Moreover, a longer lipid chain length and higher concentration leads to a greater delay of pasting and gelatinization of the starch suspension, which results in a harder cooked rice texture, while a short chain has the opposite effect (41). The ratio of AP1 to AP2 fractions is usually used as an index indicating the extent of amylopectin branching, where a higher ratio represents a higher degree of starch branching (42). In this study, the AP1/AP2 showed a positive correlation with the taste value of parental lines, but hybrid combinations showed no such correlation.

The present study indicated that the hybrid rice grains had distinct and separate ASVs. Moreover, DSC revealed two-peak thermal curves for most of the hybrid rice varieties, especially when there were obvious differences in GT between the parental lines. This is mainly because of the segregation of *ALK* alleles in the F_2_ generation. Therefore, parental lines carrying the same *ALK* allele should be selected to achieve consistent gelatinization during the breeding of quality hybrid rice. However, there are some special cases in which two-peak thermal curves still exist for certain hybrids with *ALK*^*c*^/*ALK*^*c*^ combinations (Supplementary Table 3). Moreover, the ASVs also showed an inconsistent degree of gelatinization in some hybrid rice combinations with *ALK*^*b*^/*ALK*^*b*^ (Figure 2A1). As GT is a complex quantitative trait controlled by a major gene and polygenes, previous studies identified that *ALK* and *Wx* are diversified and cooperate in influencing GT. Additionally, *SBE3, ISA*, and *SSIV-2* were minor genes that affected GT additively. Therefore, for hybrid rice, the influence of minor genes cannot be ruled out even though the major gene (*ALK*) is homozygous.

As the eating quality of hybrid rice is affected by both parents, evaluating the quality traits of hybrid lines from different kinds of parents may allow us to derive the most appropriate combination of phenotypes to achieve high quality, which will definitely improve the efficiency of the quality breeding of hybrid rice. Here, analyses of the correlated physicochemical properties between the hybrid combinations and their parental lines revealed that the eating quality traits of the hybrid combinations were determined by both parents. The sterile lines determined the overall eating quality characteristics of the hybrid combinations. Therefore, the quality characteristics of sterile lines should be considered first. For the same sterile line, there were some correlations between the hybrid combinations and restorer lines in terms of taste value, RVA breakdown and setback values, AAC, and cooked rice hardness and stickiness. Moreover, analysis of the starch fine structure in hybrid rice combinations and their restorer lines demonstrated positive correlations between them in terms of AM and AP1. Therefore, excluding AAC, the restorer lines with high breakdown values, AP1, and cooked rice stickiness that have comparatively low setback values, AM, and cooked rice hardness should be considered comprehensively.

In this study, we found that the allelic combination of *Wx*^*b*^/*Wx*^*b*^ contributed greatly to the eating quality traits of hybrid rice. This is mainly because *the Wx*^*b*^ allele is a low-amylose allele with soft texture (43, 44). Moreover, when the *Wx*^*b*^ allele was homozygous in the hybrid combinations, the AAC and other related traits of each grain tended to be more homogeneous, generally improving taste. The allele *Wx*^*a*^ has a higher amylose content and a harder cooked rice texture (44); furthermore, the overall taste value of the *Wx*^*a*^/*Wx*^*a*^ hybrid rice was lower, even when no genetic segregation occurred in the *Wx* locus of hybrid F_2_ *Wx*^*lv*^ which is responsible for low viscosity and high ACC (35). If either parent carried *Wx*^*lv*^, the overall taste value of the hybrid combinations was relatively low. *Wx*^*a*^ and *Wx*^*b*^ are the two most common alleles in the parental lines. We found obvious differences in the overall taste value between hybrid combinations with *Wx*^*a*^/*Wx*^*b*^ and *Wx*^*b*^/*Wx*^*a*^, and the overall taste value of some *Wx*^*a*^/*Wx*^*b*^ hybrids was higher than that in *Wx*^*b*^/*Wx*^*a*^ hybrids. In theory, regardless of whether the hybrid carried *Wx*^*b*^/*Wx*^*a*^ or *Wx*^*a*^/*Wx*^*b*^, the endosperm of the hybrid combinations included four genotypes: *Wx*^*b*^/*Wx*^*b*^/*Wx*^*b*^, *Wx*^*b*^/*Wx*^*b*^/*Wx*^*a*^, *Wx*^*b*^/*Wx*^*a*^/*Wx*^*a*^, and *Wx*^*a*^/*Wx*^*a*^/*Wx*^*a*^, and the ratio of genetic separation of these four genotypes was 1:1:1:1. This indicated that the genetic effects of any other genes on the eating quality traits of hybrid rice should be further analyzed. This also reminds us that a hybrid combination with a superior taste value can be obtained even when the parental lines carry different *Wx* genotypes.

In summary, this study (i) determined the physicochemical properties and starch molecular structures influencing the eating quality of hybrid rice, (ii) analyzed the relationship between hybrid combinations and parental lines in terms of the related physicochemical properties and starch molecular structures, and (iii) analyzed the genetic effects of different allelic combinations of *Wx* or *ALK* on the eating quality traits of hybrid rice, which provides a theoretical basis to improve the eating quality of *indica* hybrid rice.

## Data Availability Statement

The original contributions generated in the study are included in the article/Supplementary Materials, further inquiries can be directed to the corresponding authors.

## Author Contributions

BZ and YX: conceptualization, methodology, and supervision. WT: methodology and resources. YP: investigation, formal analysis, and writing—original draft. BM and YS: resources and investigation. TW and LH: investigation. YH, LT, and YL: validation. YP and BM contributed equally to this work. All authors contributed to the article and approved the submitted version.

## Conflict of Interest

The authors declare that the research was conducted in the absence of any commercial or financial relationships that could be construed as a potential conflict of interest.

## Abbreviations

AAC: apparent amylose content
ASV: alkali spreading value
AP1: short-branch amylopectin chains
AP2: long-branch amylopectin chains
AM: amylose
BDV: breakdown viscosity
CPV: cool paste viscosity
DSC: differential scanning calorimeter
GC: gel consistency
GPC: gel permeation chromatography
PT: pasting temperature
PC: protein content
ΔH: enthalpy of gelatinization
PKV: peak viscosity
RVA: rapid visco analyzer
SBV: setback viscosity.
