# Corrigendum: Correlations Between Parental Lines and *Indica* Hybrid Rice in Terms of Eating Quality Traits

**DOI:** 10.3389/fnut.2021.663504

**Published:** 2021-02-23

**Authors:** Yan Peng, Bigang Mao, Changquan Zhang, Ye Shao, Tianhao Wu, Liming Hu, Yuanyi Hu, Li Tang, Yaokui Li, Bingran Zhao, Wenbang Tang, Yinghui Xiao

**Affiliations:** ^1^College of Agronomy, Hunan Agricultural University, Changsha, China; ^2^State Key Laboratory of Hybrid Rice, Hunan Hybrid Rice Research Center, Changsha, China; ^3^Longping Graduate School, Hunan University, Changsha, China; ^4^Jiangsu Key Laboratory of Crop Genomics and Molecular Breeding, Jiangsu Key Laboratory of Crop Genetics and Physiology, Jiangsu Co-Innovation Center for Modern Production Technology of Grain Crops, College of Agriculture, Yangzhou University, Yangzhou, China

**Keywords:** eating quality, hybrid rice, parental lines, physicochemical character, starch molecular structure

In the original article, authors Yan Peng and Bigang Mao were not listed as sharing co-first authorship.

The authors apologize for this error and state that this does not change the scientific conclusions of the article in any way. The original article has been updated.

