# Supplementary figures and images for: Correlations Between Parental Lines and Indica Hybrid Rice in Terms of Eating Quality Traits
Source: Front Nutr. 2021 Jan 7;7:583997. doi: 10.3389/fnut.2020.583997 (PMC7817974; doi:10.3389/fnut.2020.583997)

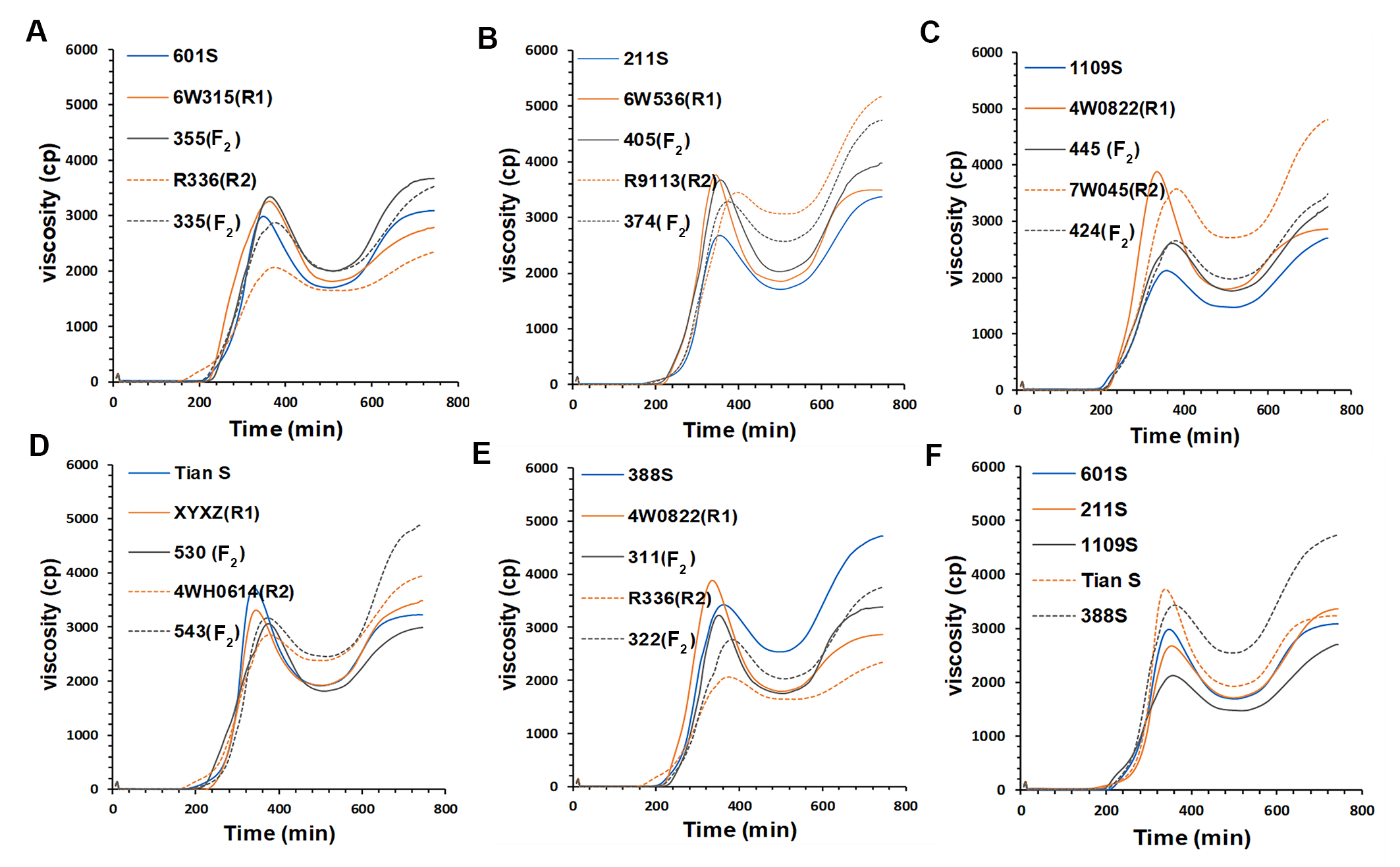

Supplement: Supplementary file 1 [file Image_1.tif]

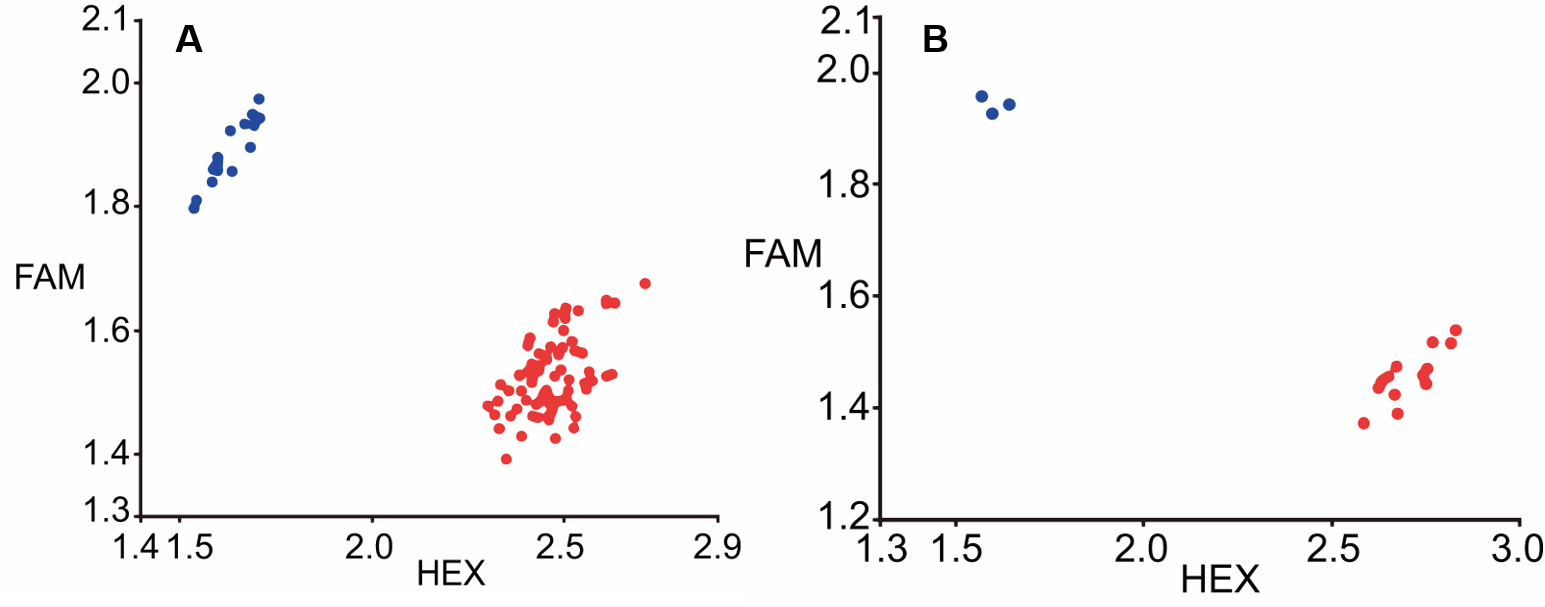

Supplement: Supplementary file 2 [file Image_2.tif]

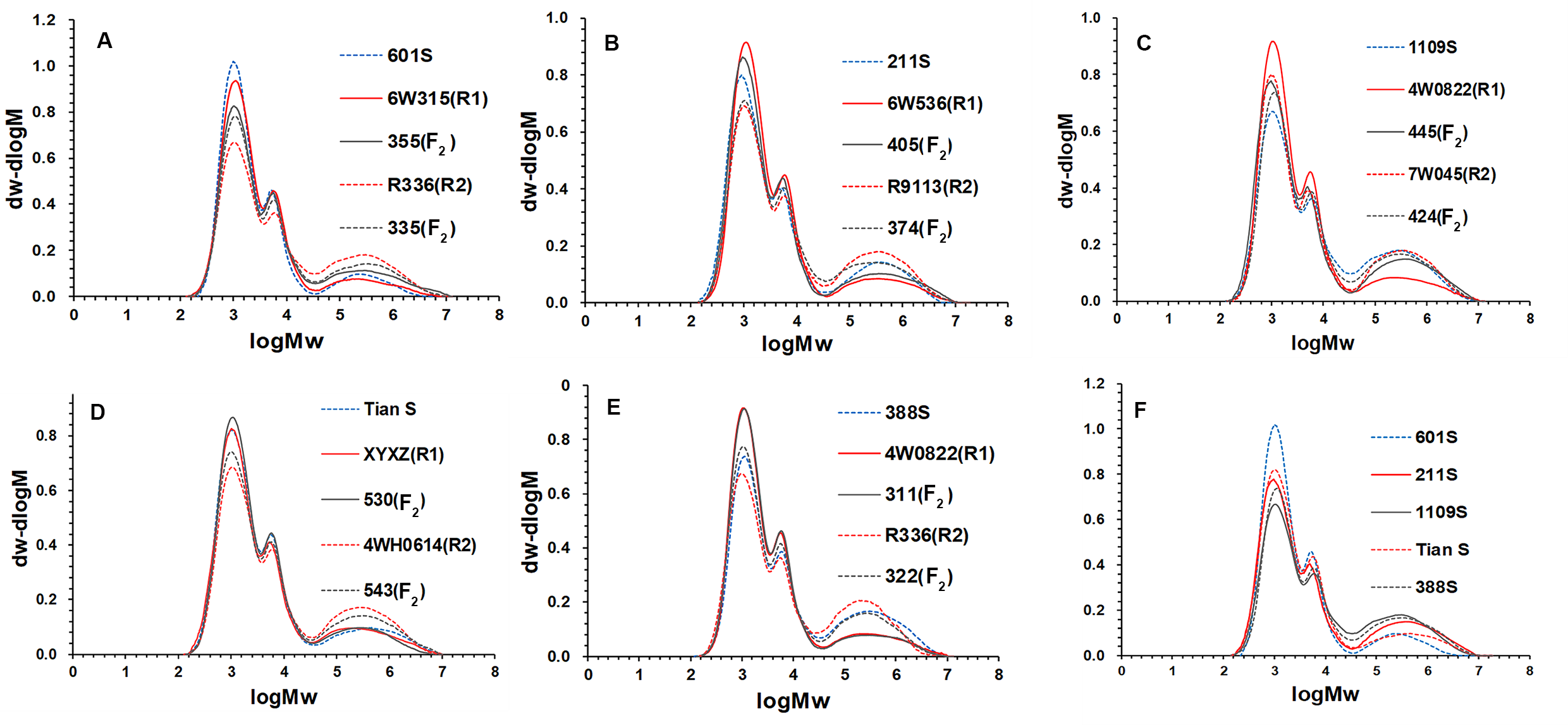

Supplement: Supplementary file 3 [file Image_3.tif]
