# Supplementary material for: Correlations Between Parental Lines and Indica Hybrid Rice in Terms of Eating Quality Traits
Source: Front Nutr. 2021 Jan 7;7:583997. doi: 10.3389/fnut.2020.583997 (PMC7817974; doi:10.3389/fnut.2020.583997)
Supplement: Supplementary file 4 [file Table_1.docx]

**Table S1.** Statistical values of physicochemical properties of hybrid rice.

|  | Taste value | BDV | SBV | PT | PKV | CPV | Hardness | Stickiness | AAC | GC | PC | LWR | CD |
| --- | --- | --- | --- | --- | --- | --- | --- | --- | --- | --- | --- | --- | --- |
| Number | 403 | 381 | 381 | 381 | 381 | 381 | 398 | 398 | 340 | 327 | 276 | 351 | 346 |
| Mean value | 78 | 1,125 | 1,303 | 80 | 2,901 | 3,074 | 3.13 | 0.16 | 17 | 48 | 8.2 | 3.2 | 2.4 |
| Minimum value | 54 | 363 | 847 | 72 | 2,142 | 2,334 | 1.43 | 0.01 | 9 | 11 | 6.8 | 2.8 | 0.08 |
| Maximum value | 90 | 1,958 | 2,357 | 89 | 3,601 | 4,316 | 6.43 | 0.79 | 29 | 123 | 10.9 | 3.8 | 12.4 |
| Median | 80 | 1,149 | 1,230 | 81 | 2,920 | 3,004 | 2.93 | 0.16 | 16 | 50 | 8.2 | 3.2 | 1.9 |
| Skewness | -0.74 | -0.11 | 1.00 | -0.64 | -0.28 | 0.75 | 0.88 | 1.07 | 0.81 | 0.47 | 0.74 | 0.50 | 1.66 |

BDV, SBV, PT, PKV, CPV, AC, GC, PC, LWR and CD correspond to breakdown value, setback value, pasting temperature, peak viscosity, final viscosity, amylose content, gel consistency, protein content, length-to-width ratio and chalkiness degree, respectively.
