# Supplementary material for: Correlations Between Parental Lines and Indica Hybrid Rice in Terms of Eating Quality Traits
Source: Front Nutr. 2021 Jan 7;7:583997. doi: 10.3389/fnut.2020.583997 (PMC7817974; doi:10.3389/fnut.2020.583997)
Supplement: Supplementary file 5 [file Table_2.docx]

**Table S2.** correlation between physicochemical properties and taste values of hybrid rice.

|  | Taste value | BDV | SBV | PT | PKV | CPV | Hardness | Stickiness | AAC | GC | PC | LWR | CD |
| --- | --- | --- | --- | --- | --- | --- | --- | --- | --- | --- | --- | --- | --- |
| Taste value | 1.00 | 0.68** | -0.70** | -0.08 | 0.60** | -0.37** | -0.77** | 0.62** | -0.61** | 0.57** | -0.42** | -0.13* | -0.10 |
| BDV | 0.68** | 1.00 | -0.68** | 0.15** | 0.78** | -0.43** | -0.60** | 0.60** | -0.64** | 0.60** | 0.10 | 0.05 | -0.24** |
| SBV | -0.70** | -0.68** | 1.00 | -0.01 | -0.52** | 0.50** | 0.62** | -0.62** | 0.63** | -0.62** | -0.16* | 0.05 | 0.11 |
| PT | -0.08 | 0.15** | -0.01 | 1.00 | 0.01 | -0.11* | -0.04 | 0.02 | -0.12* | -0.09 | -0.15 | 0.00 | 0.11 |
| PKV | 0.60** | 0.78** | -0.52** | 0.01 | 1.00 | -0.19** | -0.48** | 0.48** | -0.53** | 0.50** | 0.04 | 0.03 | -0.23** |
| CPV | -0.37** | -0.43** | 0.50** | -0.11* | -0.19** | 1.00 | 0.35** | -0.36** | 0.40** | -0.33** | -0.18* | 0.04 | 0.16** |
| Hardness | -0.77** | -0.60** | 0.62** | -0.04 | -0.48** | 0.35** | 1.00 | -0.61** | 0.59** | -0.46** | 0.10 | 0.07 | 0.13* |
| Stickiness | 0.62** | 0.60** | -0.62** | 0.02 | 0.48** | -0.36** | -.061** | 1.00 | -0.50** | 0.51** | 0.39 | -0.06 | -0.17** |
| AAC | -0.61** | -0.64** | 0.63** | -0.12* | -0.53** | 0.40** | 0.59** | -0.50** | 1.00 | -0.44** | -0.18* | 0.02 | 0.13* |
| GC | 0.57** | 0.60** | -0.62** | -0.09 | 0.50** | -0.33** | -0.46** | 0.51** | -0.44** | 1.00 | 0.16* | 0.00 | -0.20** |
| PC | 0.19* | 0.10 | -0.16* | -0.15 | 0.04 | -0.18* | -0.09 | 0.14 | -0.18* | 0.16* | 1.00 | -0.10 | 0.05 |
| LWR | -0.13* | 0.05 | 0.05 | 0.00 | 0.03 | 0.04 | 0.07 | -0.06 | 0.02 | 0.00 | -0.10 | 1.00 | -0.19** |
| CD | -0.10 | -0.24** | 0.12 | 0.11 | -0.23** | 0.16** | 0.13* | -0.17** | 0.13* | -0.20** | 0.05 | -0.19** | 1.00 |

*Correlations significant at *P* < 0.05; **Correlations significant at *P* < 0.01.
