# Supplementary material for: Correlations Between Parental Lines and Indica Hybrid Rice in Terms of Eating Quality Traits
Source: Front Nutr. 2021 Jan 7;7:583997. doi: 10.3389/fnut.2020.583997 (PMC7817974; doi:10.3389/fnut.2020.583997)
Supplement: Supplementary file 6 [file Table_3.docx]

**Table S3**. Relationships of *T*_p_ among hybrid combinations and parental lines.

| Parents | Hybrid  combinations | Genotype (*ALK*) |  | *T*_p_ (°C) | *T*_p1_ (°C) | *T*_p2_ (°C) |
| --- | --- | --- | --- | --- | --- | --- |
| 211S |  | *ALK^b^* |  | 67.2±0.3^a^ |  |  |
|  | 380 | *ALK^b^ / ALK^b^* |  | 68.5±0.2^b^ |  |  |
| 5W0454 (R) |  | *ALK^b^* |  | 69.3±0.3^c^ |  |  |
| 211S |  | *ALK^b^* |  | 67.2±0.3^a^ |  |  |
|  | 374 | *ALK^b^ / ALK^b^* |  | 69.5±0.5^b^ |  |  |
| R9113 (R) |  | *ALK^b^* |  | 67.3±0.3^a^ |  |  |
| 211S |  | *ALK^b^* |  | 67.2±0.3^a^ |  |  |
|  | 397 | *ALK^b^ / ALK^c^* |  |  | 71.5±0.2^b^ | 79.4±0.4^c^ |
| 6W315 (R) |  | *ALK^c^* |  | 79.5±0.2^c^ |  |  |
| 211S |  | *ALK^b^* |  | 67.2±0.3^a^ |  |  |
|  | 405 | *ALK^b^ / ALK^c^* |  |  | 69.5±0.5^b^ | 77.8±0.2^c^ |
| 6W536 (R) |  | *ALK^c^* |  | 78.8±0.4^d^ |  |  |
| 388S |  | *ALK^c^* |  | 75.5±0.4^c^ |  |  |
|  | 322 | *ALK^c^ / ALK^b^* |  |  | 68.0±0.5^b^ | 78.1±0.2^d^ |
| R336 (R) |  | *ALK^b^* |  | 65.6±0.3^a^ |  |  |
| 388S |  | *ALK^c^* |  | 75.5±0.4^c^ |  |  |
|  | 313 | *ALK^c^ / ALK^b^* |  |  | 69.5±0.5^b^ | 78.7±0.5^d^ |
| 4WH0614 (R) |  | *ALK^b^* |  | 67.1±0.2^a^ |  |  |
| 388S |  | *ALK^c^* |  | 75.5±0.4^a^ |  |  |
|  | 311 | *ALK^c^ / ALK^c^* |  | 80.0±0.1^c^ |  |  |
| 4W0822 (R) |  | *ALK^c^* |  | 78.5±0.6^b^ |  |  |
| 388S |  | *ALK^c^* |  | 75.5±0.4^c^ |  |  |
|  | 304 | *ALK^c^ / ALK^b^* |  |  | 67.5±0.4^a^ | 78.8±0.8^d^ |
| 7W493 (R) |  | *ALK^b^* |  | 69.0±0.5^b^ |  |  |
| 1109S |  | *ALK^c^* |  | 76.8±0.2^a^ |  |  |
|  | 445 | *ALK^c^ / ALK^c^* |  | 79.3±0.3^c^ |  |  |
| 4W0822 (R) |  | *ALK^c^* |  | 78.5±0.6^b^ |  |  |
| 1109S |  | *ALK^c^* |  | 76.8±0.2^a^ |  |  |
|  | 424 | *ALK^c^ / ALK^c^* |  | 81.1±0.4^b^ |  |  |
| 7W045 (R) |  | *ALK^c^* |  | 76.5±0.3^a^ |  |  |
| 601S |  | *ALK^c^* |  | 76.5±0.3^c^ |  |  |
|  | 335 | *ALK^c^ / ALK^b^* |  |  | 69.6±0.8^b^ | 78.0±0.3^d^ |
| R336 (R) |  | *ALK^b^* |  | 65.6±0.3^a^ |  |  |
| 601S |  | *ALK^c^* |  | 76.5±0.3^c^ |  |  |
|  | 343 | *ALK^c^ / ALK^b^* |  |  | 68.5±0.6^b^ | 76.7±0.4^c^ |
| 4WH0614 (R) |  | *ALK^b^* |  | 67.1±0.2^a^ |  |  |
| 601S |  | *ALK^c^* |  | 76.5±0.3^b^ |  |  |
|  | 354 | *ALK^c^ / ALK^c^* |  | 80.3±0.4^c^ |  |  |
| 6W1003 (R) |  | *ALK^c^* |  | 75.7±0.5^a^ |  |  |
| 601S |  | *ALK^c^* |  | 76.5±0.3^c^ |  |  |
|  | 350 | *ALK^c^ / ALK^b^* |  |  | 74.0±0.4^b^ | 80.5±0.3^d^ |
| 5WH125 (R) |  | *ALK^b^* |  | 69.4±0.5^a^ |  |  |
| 601S |  | *ALK^c^* |  | 76.5±0.3^b^ |  |  |
|  | 340 | *ALK^c^ / ALK^c^* |  |  | 70.5±0.2^a^ | 78.6±0.5c |
| 4W0822 (R) |  | *ALK^c^* |  | 78.5±0.6^c^ |  |  |
| 601S |  | *ALK^c^* |  | 76.5±0.3^a^ |  |  |
|  | 355 | *ALK^c^ / ALK^c^* |  | 81.2±0.3^c^ |  |  |
| 6W315 (R) |  | *ALK^c^* |  | 79.5±0.2^b^ |  |  |
| Tian S |  | *ALK^b^* |  | 69.9±0.5^b^ |  |  |
|  | 543 | *ALK^b^ / ALK^b^* |  | 69.2±0.5^b^ |  |  |
| 4WH0614 (R) |  | *ALK^b^* |  | 67.1±0.2^a^ |  |  |
| Tian S |  | *ALK^b^* |  | 69.9±0.5^b^ |  |  |
|  | 520 | *ALK^b^ / ALK^c^* |  |  | 69.0±0.4^a^ | 76.6±0.3c |
| 7W045 (R) |  | *ALK^c^* |  | 76.5±0.3^c^ |  |  |
| Tian S |  | *ALK^b^* |  | 69.9±0.5^a^ |  |  |
|  | 555 | *ALK^b^ / ALK^c^* |  |  | 71.5±0.4^b^ | 80.3±0.4^d^ |
| 4W0822 (R) |  | *ALK^c^* |  | 78.5±0.6^c^ |  |  |
| Tian S |  | *ALK* *^b^* |  | 69.9±0.4^a^ |  |  |
|  | 542 | *ALK^b^ / ALK^c^* |  |  | 71.0±0.5^b^ | 78.4±0.4^c^ |
| 5W0648 (R) |  | *ALK^c^* |  | 78.4±0.5^c^ |  |  |
| Tian S |  | *ALK^b^* |  | 69.9±0.5^a^ |  |  |
|  | 530 | *ALK^b^ / ALK^c^* |  |  | 71.4±0.2^b^ | 80.0±0.4^c^ |
| XYXZ (R) |  | *ALK^c^* |  | 80.5±0.3^c^ |  |  |
| Tian S |  | *ALK^b^* |  | 69.9±0.5^a^ |  |  |
|  | 540 | *ALK^b^ / ALK^b^* |  | 71.1±0.2^b^ |  |  |
| 5W1009 (R) |  | *ALK^b^* |  | 69.6±0.4^a^ |  |  |

*T*_p1_, peak temperature of peak 1; *T*_p2_, peak temperature of peak 2. Different superscripted letters in the same column indicate significant differences (*p* < 0.05).
