# Supplementary material for: Correlations Between Parental Lines and Indica Hybrid Rice in Terms of Eating Quality Traits
Source: Front Nutr. 2021 Jan 7;7:583997. doi: 10.3389/fnut.2020.583997 (PMC7817974; doi:10.3389/fnut.2020.583997)
Supplement: Supplementary file 7 [file Table_4.docx]

**Table S4**. GPC parameters of hybrid combinations and parental lines

| Parents | Hybrids | | Taste value | AAC(%) | GPC peak area (%) | | | Area ratio |  |
| --- | --- | --- | --- | --- | --- | --- | --- | --- | --- |
|  |  |  |  |  | AP1/AP | AP2/AP | AM/  (AM+AP1+AP2) | AP1/AP2 |  |
| 211S |  | | 75.30±0.80^b^ | 18.10±0.22^b^ | 60.55±0.03^b^ | 21.32±0.13^b^ | 18.13±0.10^b^ | 2.84±0.02^b^ |  |
|  | 380 | | 63.47±0.65^a^ | 23.27±0.20^c^ | 53.85±0.01^a^ | 21.69±0.12^c^ | 24.46±0.12^c^ | 2.48±0.01^a^ |  |
| 5W0454 (R) |  | | 86.37±0.65^c^ | 16.22±0.24^a^ | 63.59±0.20^c^ | 20.88±0.11^a^ | 15.52±0.10^a^ | 3.05±0.02^c^ |  |
| 211S |  | | 75.30±0.80^c^ | 18.10±0.22 ^a^ | 60.55±0.03^c^ | 21.32±0.13^b^ | 18.13±0.10^a^ | 2.84±0.02^c^ |  |
|  | 374 | | 68.10±0.60^b^ | 22.37±0.15^b^ | 55.47±0.03^b^ | 22.35±0.06^c^ | 22.18±0.08^b^ | 2.48±0.01^a^ |  |
| R9113 (R) |  | | 64.70±1.10^a^ | 25.09±0.08^c^ | 54.34±0.14^a^ | 19.76±0.07^a^ | 25.89±0.08^c^ | 2.75±0.02^b^ |  |
| 211S |  | | 75.30±0.80^a^ | 18.10±0.22^c^ | 60.55±0.03^a^ | 21.32±0.13^a^ | 18.13±0.10^c^ | 2.84±0.02^b^ |  |
|  | 397 | | 83.63±0.45^b^ | 16.24±0.22^b^ | 62.20±0.27^b^ | 23.03±0.16^c^ | 14.77±0.11^b^ | 2.70±0.03^a^ |  |
| 6W315 (R) |  | | 88.37±0.55^c^ | 15.28±0.16^a^ | 67.10±0.18^c^ | 22.32±0.06^b^ | 10.57±0.13^a^ | 3.01±0.02^c^ |  |
| 211S |  | | 75.30±0.80^a^ | 18.10±0.22^c^ | 60.55±0.03^a^ | 21.32±0.13^a^ | 18.13±0.10^c^ | 2.84±0.02^a^ |  |
|  | 405 | | 85.10±0.50^b^ | 17.21±0.21^b^ | 62.47±0.07^b^ | 21.72±0.14^b^ | 15.80±0.07^b^ | 2.88±0.02^b^ |  |
| 6W536 (R) |  | | 86.50±0.40^c^ | 16.23±0.26^a^ | 64.84±0.05^c^ | 21.85±0.06^b^ | 13.31±0.11^a^ | 2.97±0.01^c^ |  |
| 388S |  | | 65.70±0.60^b^ | 23.20±0.15^b^ | 53.12±0.17^b^ | 22.29±0.09^b^ | 24.59±0.09^b^ | 2.38±0.02^a^ |  |
|  | 322 | | 64.67±0.66^b^ | 22.28±0.10^a^ | 55.42±0.09^c^ | 22.39±0.15^b^ | 22.19±0.06^a^ | 2.48±0.02^c^ |  |
| R336 (R) |  | | 60.43±1.05^a^ | 26.13±0.11^c^ | 51.11±0.02^a^ | 20.99±0.09^a^ | 27.91±0.07^c^ | 2.44±0.01^b^ |  |
| 388S |  | | 65.70±0.60^b^ | 23.20±0.15^b^ | 53.12±0.17^a^ | 22.29±0.09^b^ | 24.59±0.09^b^ | 2.38±0.02^a^ |  |
|  | 313 | | 68.33±0.55^c^ | 21.33±0.14^a^ | 57.52±0.24^c^ | 22.09±0.18^b^ | 20.39±0.07^a^ | 2.60±0.03^b^ |  |
| 4WH0614 (R) |  | | 61.90±0.50^a^ | 25.37±0.21^c^ | 54.33±0.24^b^ | 20.22±0.16^a^ | 25.45±0.09^c^ | 2.69±0.03^c^ |  |
| 388S |  | | 65.70±0.60^a^ | 23.20±0.15^c^ | 53.12±0.17^a^ | 22.29±0.09^a^ | 24.59±0.09^c^ | 2.38±0.02^a^ |  |
|  | 311 | | 85.90±1.11^b^ | 15.33±0.18^b^ | 64.37±0.03^b^ | 23.04±0.07^b^ | 12.59±0.10^b^ | 2.79±0.01^b^ |  |
| 4W0822 (R) |  | | 86.50±0.40^b^ | 13.25±0.25^a^ | 65.19±0.18^c^ | 23.38±0.08^c^ | 11.43±0.10 ^a^ | 2.79±0.02^b^ |  |
| 388S |  | | 65.70±0.60^a^ | 23.20±0.15^c^ | 53.12±0.17^a^ | 22.29±0.09^b^ | 24.59±0.09^c^ | 2.38±0.02^a^ |  |
|  | 304 | | 86.77±0.55^c^ | 16.29±0.08^a^ | 61.28±0.21^c^ | 23.68±0.16^c^ | 15.03±0.06^a^ | 2.59±0.03^b^ |  |
| 7W493 (R) |  | | 80.13±0.55^b^ | 18.09±0.17^b^ | 58.84±0.12^b^ | 20.18±0.05^a^ | 20.97±0.08^b^ | 2.92±0.01^c^ |  |
| 1109S |  | | 62.33±0.75^a^ | 26.13±0.20^c^ | 51.80±0.22^a^ | 21.77±0.06^b^ | 26.43±0.16^c^ | 2.38±0.02^a^ |  |
|  | 445 | | 79.26±0.56^b^ | 21.22±0.22^b^ | 56.40±0.23^b^ | 23.53±0.06^a^ | 20.06±0.18^b^ | 2.40±0.02^c^ |  |
| 4W0822 (R) |  | 86.50±0.40^c^ | | 13.25±0.25^a^ | 65.19±0.18^c^ | 23.38±0.08^c^ | 11.43±0.10^a^ | 2.79±0.02^b^ |  |
| 1109S |  | 62.33±0.75^a,b^ | | 26.13±0.20^c^ | 51.80±0.22^a^ | 21.77±0.06^b^ | 26.43±0.16^b^ | 2.38±0.02^a^ |  |
|  | 424 | | 61.63±0.45^a^ | 25.01±0.09^b^ | 52.65±0.04^b^ | 21.04±0.16^a^ | 26.31±0.12^b^ | 2.50±0.02^b^ |  |
| 7W045 (R) |  | | 63.40±0.70^b^ | 24.33±0.21^a^ | 54.52±0.24^c^ | 21.11±0.10^a^ | 24.36±0.15^a^ | 2.58±0.02^c^ |  |
| 601S |  | | 87.37±0.45^c^ | 13.22±0.26^a^ | 68.99±0.04^c^ | 20.65±0.11^a^ | 10.36±0.15^a^ | 3.34±0.02^c^ |  |
|  | 335 | | 70.17±0.55^b^ | 23.37±0.26^b^ | 56.45±0.21^b^ | 22.39±0.06^c^ | 21.16±0.15^b^ | 2.52±0.02^b^ |  |
| R336 (R) |  | | 60.43±1.05^a^ | 26.13±0.11^c^ | 51.11±0.02^a^ | 20.99±0.09^b^ | 27.91±0.07^c^ | 2.44±0.01^a^ |  |
| 601S |  | | 87.37±0.45^c^ | 13.22±0.26 ^a^ | 68.99±0.04^c^ | 20.65±0.11^b^ | 10.36±0.15^a^ | 3.34±0.02^c^ |  |
|  | 343 | | 70.37±0.70^b^ | 22.33±0.15^b^ | 55.86±0.27^b^ | 22.62±0.11^c^ | 21.52±0.17^b^ | 2.47±0.02^a^ |  |
| 4WH0614 (R) |  | | 61.90±0.50^a^ | 25.37±0.21^c^ | 54.33±0.24^a^ | 20.22±0.16^a^ | 25.45±0.09^c^ | 2.69±0.03^b^ |  |
| 601S |  | | 87.37±0.45^c^ | 13.22±0.26^a^ | 68.99±0.04^c^ | 20.65±0.11^b^ | 10.36±0.15^a^ | 3.34±0.02^c^ |  |
|  | 354 | | 71.63±0.50^b^ | 16.23±0.10^b^ | 61.56±0.01^b^ | 23.47±0.15^c^ | 14.96±0.16^b^ | 2.62±0.02^a^ |  |
| 6W1003 (R) |  | | 66.50±0.40^a^ | 23.18±0.36^c^ | 54.31±0.06^a^ | 19.19±0.11^a^ | 26.50±0.17^c^ | 2.83±0.01^b^ |  |
| 601S |  | | 87.37±0.45^b^ | 13.22±0.26^a^ | 68.99±0.04 ^b^ | 20.65±0.11^b^ | 10.36±0.15^a^ | 3.34±0.02^c^ |  |
|  | 350 | | 71.13±0.67^a^ | 15.47±0.20^b^ | 64.68±0.03^a^ | 21.76±0.16^c^ | 13.56±0.13^b^ | 2.97±0.02^a^ |  |
| 5WH125 (R) |  | | 86.73±0.25^b^ | 15.54±0.25 ^b^ | 64.77±0.20^a^ | 19.98±0.13^a^ | 15.25±0.07^c^ | 3.24±0.03^b^ |  |
| 601S |  | | 87.37±0.45^b^ | 13.22±0.26^a^ | 68.99±0.04^c^ | 20.65±0.11^a^ | 10.36±0.15^a^ | 3.34±0.02^c^ |  |
|  | 340 | | 84.37±0.75^a^ | 16.38±0.12^b^ | 58.87±0.14^a^ | 24.17±0.06^c^ | 16.96±0.08^c^ | 2.44±0.01^a^ |  |
| 4W0822 (R) |  | | 86.50±0.40^b^ | 13.25±0.25^a^ | 65.19±0.18^b^ | 23.38±0.08^b^ | 11.43±0.10^b^ | 2.79±0.02^b^ |  |
| 601S |  | | 87.37±0.45^b^ | 13.22±0.26^a^ | 68.99±0.04^c^ | 20.65±0.11^a^ | 10.36±0.15^a^ | 3.34±0.02^c^ |  |
|  | 355 | | 85.40±0.50^a^ | 15.13±0.22^b^ | 58.77±0.17^a^ | 25.59±0.12^c^ | 16.64±0.05^c^ | 2.26±0.02^a^ |  |
| 6W315 (R) |  | | 88.40±0.70^b^ | 15.35±0.23^b^ | 67.11±0.13^b^ | 22.32±0.05^b^ | 10.57±0.08^b^ | 3.01±0.01^b^ |  |
| Tian S |  | | 85.13±0.35^c^ | 15.28±0.19^a^ | 63.30±0.16^c^ | 21.61±0.1^b^ | 15.09±0.06^a^ | 2.93±0.02^c^ |  |
|  | 543 | | 67.63±0.55^b^ | 21.36±0.16^b^ | 56.60±0.01^b^ | 22.12±0.15^c^ | 21.27±0.16^b^ | 2.56±0.02^a^ |  |
| 4WH0614 (R) |  | | 61.90±0.50^a^ | 25.37±0.21^c^ | 54.33±0.24^a^ | 20.22±0.16^a^ | 25.45±0.09^c^ | 2.69±0.03^b^ |  |
| Tian S |  | | 85.13±0.35^c^ | 15.28±0.19^a^ | 63.30±0.16^c^ | 21.61±0.1^b^ | 15.09±0.06^a^ | 2.93±0.02^b^ |  |
|  | 520 | | 68.77±0.35^b^ | 25.02±0.11^c^ | 55.36±0.02^b^ | 21.61±0.08^b^ | 23.03±0.10^b^ | 2.56±0.01^a^ |  |
| 7W045 (R) |  | | 63.37±0.45^a^ | 24.30±0.15^b^ | 54.53±0.04^a^ | 21.11±0.12^a^ | 24.36±0.08^c^ | 2.58±0.02^a^ |  |
| Tian S |  | | 85.13±0.35^a^ | 15.28±0.19^b^ | 63.30±0.16^a^ | 21.61±0.10^a^ | 15.09±0.06^c^ | 2.93±0.02^c^ |  |
|  | 555 | | 85.17±0.55^a^ | 15.33±0.14 ^b^ | 63.67±0.01^b^ | 23.14±0.06^b^ | 13.19±0.06^b^ | 2.75±0.01^a^ |  |
| 4W0822 (R) |  | | 86.50±0.40^b^ | 13.25±0.25^a^ | 65.19±0.18^c^ | 23.38±0.08^c^ | 11.43±0.10^a^ | 2.79±0.02^b^ |  |
| Tian S |  | | 85.13±0.35^a^ | 15.28±0.19^b^ | 63.30±0.16^b^ | 21.61±0.10^a^ | 15.09±0.06^b^ | 2.93±0.02^b^ |  |
|  | 542 | | 85.37±0.55^a^ | 16.28±0.28^c^ | 63.09±0.02^a^ | 21.82±0.10^a^ | 15.08±0.08^b^ | 2.89±0.01^a^ |  |
| 5W0648 (R) |  | | 91.67±0.95^b^ | 14.53±0.16^a^ | 67.38±0.04^c^ | 21.76±0.12^a^ | 10.86±0.09^a^ | 3.10±0.02^c^ |  |
| Tian S |  | | 85.13±0.35^b^ | 15.28±0.19^a^ | 63.30±0.16^a^ | 21.61±0.10^b^ | 15.09±0.06^c^ | 2.93±0.02^b^ |  |
|  | 530 | | 85.67±0.75^b^ | 15.26±0.18^a^ | 63.72±0.16^b^ | 22.82±0.09^c^ | 13.46±0.08^a^ | 2.79±0.02^a^ |  |
| XYXZ (R) |  | | 82.27±0.35^a^ | 16.13±0.10^b^ | 64.58±0.07^c^ | 21.06±0.08^a^ | 14.36±0.15^b^ | 3.07±0.01^c^ |  |
| Tian S |  | | 85.13±0.35^b^ | 15.28±0.19^a^ | 63.30±0.16^b^ | 21.61±0.10^a^ | 15.09±0.06^b^ | 2.93±0.02^c^ |  |
|  | 540 | | 68.77±1.35^a^ | 16.07±0.06^b^ | 57.40±0.02^a^ | 23.23±0.08^c^ | 16.37±0.06^c^ | 2.47±0.01^a^ |  |
| 5W1009 (R) |  | 88.67±0.45^c^ | | 15.36±0.27^a^ | 63.27±0.33^b^ | 22.53±0.16^b^ | 14.20±0.17^a^ | 2.81±0.03^b^ |  |

AP, AP1, AP2, and AM correspond to amylopectin, short-branch amylopectin chains, long-branch amylopectin chains, and amylose, respectively. Different superscripted letters in the same column indicate significant differences (P<0.05).
